# Upcycling post-harvest biomass residues from native European *Lupinus* species: from straws and pod shells production to nutritive value and alkaloids content for ruminant animals

**DOI:** 10.3389/fnut.2023.1195015

**Published:** 2023-07-13

**Authors:** Margarida R. G. Maia, André Monteiro, Inês M. Valente, Carla Sousa, Carla Miranda, Carlos Castro, Paulo P. Cortez, Ana R. J. Cabrita, Henrique Trindade, António J. M. Fonseca

**Affiliations:** ^1^REQUIMTE, LAQV, ICBAS, School of Medicine and Biomedical Sciences, University of Porto, Porto, Portugal; ^2^Centre for the Research and Technology of Agro-Environmental and Biological Sciences (CITAB), University of Trás-os-Montes and Alto Douro, Vila Real, Portugal; ^3^REQUIMTE, LAQV, Department of Chemistry and Biochemistry, Faculty of Sciences, University of Porto, Porto, Portugal; ^4^ICBAS, School of Medicine and Biomedical Sciences, University of Porto, Porto, Portugal

**Keywords:** alkaloids, biomass residues, by-products, *Lupinus* species, nutritive value, ruminants

## Abstract

The production of *Lupinus* seeds for food and feed is increasing worldwide, which results in large amounts of post-harvest biomass residues, considered of low value and left in the field to be burned or incorporated in the soil. To valorize these agricultural wastes, this work aimed to assess their potential as an alternative feed for ruminants. Thus, the production yield, nutritive value, and alkaloid content of straws and pod shells from three native European *Lupinus* species, *L. albus* ‘Estoril’ (white), *L. angustifolius* ‘Tango’ (narrow-leafed), and *L. luteus* ‘Cardiga’ (yellow), cultivated in two locations, were evaluated. The dry matter (DM) yield of straws and pod shells were the highest for *L. albus* ‘Estoril’ (4.10 t ha^−1^) and the lowest for *L. angustifolius* ‘Tango’ (1.78 t ha^−1^), suggesting a poor adaptation of narrow-leafed lupin to the particularly dry and warm agronomic year. Despite species-specific differences, lupin biomass residues presented higher crude protein (53.0–68.9 g kg^−1^ DM) and lignin (103–111 g kg^−1^ DM) content than cereal straws usually used in ruminant feeding, thus resulting in higher metabolizable energy (6.43–6.58 MJ kg^−1^ DM) content. *In vitro* digestibility was similar among lupin species (47.7–50.6%) and higher in pod shells (53.7%) than in straws (44.6%). *Lupinus albus* ‘Estoril’ and *L. luteus* ‘Cardiga’ presented considerable amounts of alkaloids in straws (23.9 and 119 mg kg^−1^ DM) and pod shells (20.5 and 298 mg kg^−1^ DM), while no alkaloids were detected in *L. angustifolius* ‘Tango’ biomass residues. Considering the combined production of straw and pod shells per lupin species, it is anticipated that lupin biomass residues produced per ha can fulfill 85% of the energy and nearly 50% of protein requirements of a flock of 4 to 9 dry and mid-pregnancy sheep with 50 kg body weight for one year. No negative effects on small (ovine) and large (bovine) ruminant species due to alkaloids are expected, even if biomass residues are consumed at up to 85% DM intake. The large production yield along with its nutritive value unveils the potential of lupin biomass residues valorization as alternative fodder for ruminants, promoting sustainability under a circular economy approach.

## 1. Introduction

The European Union (EU) is heavily dependent on imports of vegetable protein sources, only assuring a self-sufficiency of 79% for rapeseed, 42% for sunflower, and 5% for soya (1). Annual imports account for 17 million tons of crude protein (CP), over 76% of which of soya, the most prevailing protein source for feed and food (1). Although human consumption of vegetable proteins is raising in the EU, with the market for meat and dairy alternatives growing by 14 and 11% per year, respectively, the animal feed sector is by far the most important outlet (93% by volume) (1).

Grain legumes can effectively contribute to balance the European economic trade of plant-based protein sources for feed and food, but also play a key role in sustainable agricultural intensification (2). The cultivation of legumes has several benefits, including (i) fixation of atmospheric nitrogen (N) into the soil with improvement of soil fertility and reduction of chemical fertilizers (3); (ii) improvement of soil structure and health in crop rotation cycles and intercropping, allowing the replacement of traditional fallow and increasing the productivity of the next crop cycle (3, 4), (iii) promotion of biodiversity of rural landscape (5); (iv) contribution to mitigate greenhouse gases emissions and to tackle climate change (6, 7); and (v) improvement of farming profitability and efficiency (8).

White (*Lupinus albus* L.), yellow (*Lupinus luteus* L.), and blue or narrow-leafed (*Lupinus angustifolius* L.) lupins are native European legumes well adapted to acidic, sandy soils, a trait that differentiates them from other grain legumes (9). Lupins produce grains with high protein content (up to 44% dry matter, DM, basis) (10), with high lysine content but deficient in methionine and cysteine (11). Even though the world lupin production is increasing, in Europe the production area and yields are still modest (12, 13), reflecting a difficulty of European farmers to alter the cropping systems toward a transition to legume-supported farming (14).

*Lupinus* grains are harvest when dry, resulting in large amounts of biomass residues, including stalks, leaves, and pod shells, that traditionally remain in the fields to be burned or incorporated in the soil. Although burning is still used, post-harvest biomass incorporation is the most common practice as it improves soil fertility, through the increase of organic matter, N, phosphorus (P), and other nutrients (4, 15, 16), and the promotion of soil microbial composition and diversity (17). Post-harvest biomass residues may be further valorized as animal feed, especially in more extensive systems, either by direct grazing by small ruminants, or conservation for periods of fodder scarcity (18). Lupin biomass residues may be of greater relevance for the agricultural systems of the Mediterranean region, as the prolonged periods of severe and extreme drought aggravated by climate change and the increased incidence of large fires have drastically reduced the availability of pastures for ruminant animals.

Like other legumes, lupin straws have been reported to present higher CP and neutral detergent-soluble carbohydrates content than cereal straws, with overall greater DM digestibility but lower neutral detergent fiber (NDF) digestibility, due to the higher lignin content (19–21). Lupin straws and pod shells may present an additional challenge to be used as feed due to their potential alkaloid content. In bitter narrow-leafed lupin, quinolizidine alkaloids were shown to be produced in the aerial parts of the plant and then transferred to grains (22). The content of these secondary metabolites reduces in stems and leaves with plant maturation, increasing in pods from flowering to grain formation and then decreasing as they accumulate in grains (23). Although it may be anticipated that lupin straws and pod shells present low alkaloid content, no reports were found in the literature. Thus, to effectively assess the potential of lupin biomass residues as alternative feeds for ruminant animals, alkaloids content and profile must be characterized to predict the potential exposure of ruminants to these phytochemicals that may present toxic and teratogenic effects on ruminants (24).

In this context, the present study aimed to evaluate the potential of post-harvest biomass residues of three natives European *Lupinus* sp., white (*L. albus* ‘Estoril’), narrow-leafed (*L. angustifolius* ‘Tango’), and yellow (*L. luteus* ‘Cardiga’), as alternative feeds for ruminant animals. To achieve this aim, the production, nutritive value and detailed alkaloid characterization were assessed on straws and pod shells of the three lupin cultivars harvested in two locations (Mirandela and Vila Real). The biomass residues here assessed correspond to the first sowing date (mid-September) of the field experiment presented by Monteiro et al. (25), as it was the sowing with the highest production yield of *Lupinus* grain.

## 2. Materials and methods

### 2.1. Fields location and edaphoclimatic characteristics

The experiments were conducted simultaneously in two locations, Mirandela (41.511896, −7.161595) and Vila Real (41.284747, −7.738875), in the Trás-os-Montes region, Portugal, between September 2018 and June 2019.

The soil at Mirandela was an Eutric Fluvisol from unconsolidated material with more than 1 m deep (IUSS Working Group WRB, 2015), with the following average physical–chemical properties: pH (H_2_O): 6.10; total organic matter (OM, g kg^−1^): 11.0; extractable P (mg P_2_O_5_ kg^−1^): 224.5; exchangeable aluminum (cmolc kg^−1^): 0; effective cation exchange capacity (ECEC, cmolc kg^−1^): 6.31. The soil at Vila Real was a sandy clay Dystrophic Cambisol (IUSS Working Group WRB, 2015) derived from rocks metasedimentary Paleozoic, with pH (H_2_O): 4.75, total OM (g kg^−1^): 14.0; extractable P (mg P_2_O_5_ kg^−1^): 67.0; exchangeable aluminum (cmolc kg^−1^): 0.68; ECEC (cmolc kg^−1^): 4.24. Extractable P_2_O_5_ was determined by the Egnér-Rhiem method (26).

Soils were mobilized by tillage 15 days before sowing, followed by cross scarification, thus achieving a mobilized depth of about 20 cm.

Temperatures and rainfall in Mirandela and Vila Real followed similar trends during the field experiment (i.e., September 2018 to June 2019). The average maximum and minimum temperatures recorded were higher than the historical data registered between 1971 and 2010, particularly in Vila Real. On the other hand, the precipitation was lower throughout the agronomic year compared to the historical (1971–2010) rainfall data, the year being particularly dry in Vila Real. The exceptions were the months of November and April. In these 2 months, the maximum and minimum temperatures were lower and the rainfall nearly two folds the average historical data.

### 2.2. Experimental design and treatments

The trial was designed in casualized complete blocks with four replications and two factors: location (Mirandela and Vila Real) and *Lupinus* species (*L. albus* ‘Estoril,’ *L. angustifolius* ‘Tango’ and *L. luteus* ‘Cardiga’), resulting in 12 plots by location. Plots were marked in the experimental fields, each with a rectangular section of 2.5 × 4.0 m (10 m^2^). Sowing took place on the same day (18th September) in both locations. Sowing density was set at 100 kg ha^−1^ for white lupin, 80 kg ha^−1^ for narrow-leafed, and 60 kg ha^−1^ for yellow lupin; the distance between the rows was always 0.30 m. No agricultural procedures were performed from sowing until harvesting. The cultures were exclusively rainfed. After grain maturation, on the same day in both locations (20th June), the aerial part of the plants was harvested (2 m^2^ of area), and grains, pod shells, and straws separated and weighed. Representative subsamples (*ca.* 500 g) of each biomass residue were dried in a forced-air oven at 60°C for 48 h.

### 2.3. *In vitro* digestibility

*In vitro* dry matter digestibility (DMD) and OM digestibility (OMD) of straw and pod shell samples were determined according to Tilley and Terry (27) methodology modified by Goering and Van Soest (28). Two healthy and lactating Holstein cows fitted with rumen cannula (10 cm diameter; Bar Diamond Inc., Parma, ID) were used as rumen inocula donors. Cows were housed at Vairão Agricultural Campus of School of Medicine and Biomedical Sciences, University of Porto (ICBAS-UP, Vila do Conde, Portugal), following good animal practices for care and management of the EU (Directive, 2010/63/EU). Animal procedures and methodologies were approved by the Animal Ethics Committee of ICBAS-UP, licensed by the Portuguese General Directorate for Food and Veterinary (permit #0421/000/000/2021), and performed by trained scientists (FELASA category C). Cows were fed a corn silage-based diet with forage to concentrate ratio of 65:35 (13% CP and 19% starch, DM basis) at 08:00 and 18:00 h, with unlimited access to fresh drinking water and mineral salt blocks. Ruminal fluid was collected 3 h after the morning feed, strained through four layers of gauze, and kept at 39°C under O_2_-free CO_2_. Five hundred mg of each sample were incubated in 50 mL centrifuge tubes with 25 mL buffered rumen fluid solution (1 strained rumen fluid:4 Kansas State buffer) (29), flushed with O_2_-free CO_2_, and closed with rubber stoppers fitted to a Bunsen valve. Blanks (with buffered rumen fluid and without sample) were incubated along with the straw and pod shell samples. Tubes were incubated for 48 h at 39°C in a water bath, under continuous orbital agitation. At the end of the incubation, the contents were filtered through a fritted crucible (porosity 40–100 μm, P2), and the residues were extracted in boiling neutral detergent solution (30) for 1 h. After oven drying at 103°C for 18 h, crucibles were weighed, and the *in vitro* DM digestibility was calculated as the difference between the incubated DM and the residue that remained in the crucible (undigested) DM. The samples were corrected for bacterial and residual DM by subtracting the blanks. Crucibles were further incinerated in a muffle furnace at 500°C for 3 h, and the *in vitro* OM digestibility was calculated as described for DM digestibility. Blank residues were also used to correct the OM digestibility of samples. *In vitro* digestibility was evaluated in three runs on independent days.

The proximate composition and *in vivo* metabolizable energy (ME) data of nine Portuguese legume straws, including *L. luteus* ‘Cardiga’ and *L. albus* ‘Estoril,’ reported by Abreu and Bruno-Soares (21), were used to establish an equation to estimate the lupin biomass ME content. The equation was defined as: ME (MJ kg^−1^ DM) = 8.52–0.0188 acid detergent lignin (ADL; g kg^−1^ DM) (*r*^2^ = 0.812; RSD = 0.1857).

### 2.4. Chemical analyses

#### 2.4.1. Proximate composition

Straw and pod shell samples were milled at 1-mm screen and the proximate composition was analyzed according to official methods (31). All samples were analyzed for dry matter (DM; ID 934.01), ash (ID 942.05), ether extract (EE; ID 920.39), and Kjeldahl N (ID 954.01) contents. Crude protein was calculated as Kjeldahl N × 6.25. Neutral detergent fiber (NDF; without sodium sulfite), acid detergent fiber (ADF), and ADL of straw and pod samples were analyzed; NDF and ADL were expressed exclusive of residual ash and the ADF inclusive of residual ash (30, 32). All parameters were expressed as g kg^−1^ DM. Gross energy (GE) content was determined by using an adiabatic bomb calorimeter (Werke C2000, IKA, Staufen, Germany) and expressed as MJ kg^−1^ DM.

#### 2.4.2. Alkaloid composition

Straw and pod shell alkaloids were extracted according to Magalhães et al. (33), in duplicate. Briefly, 2 g of dried sample (1-mm) was extracted with 20 mL of trichloroacetic acid (5% w/v) for 30 min by ultrasonics, and centrifuged at 250 × *g* for 15 min. The supernatant was collected, and the extraction of the residue was repeated two more times. The combined supernatant was mixed with 4 mL of sodium hydroxide (10 mol L^−1^) and subjected to liquid–liquid extraction with dichloromethane (3 × 20 mL). The organic extract was completely evaporated under reduced pressure at 40°C. The final dry residue was resuspended in 2 mL of dichloromethane for GC–MS analysis, filtered with a 0.45 μm regenerated cellulose syringe filter, and stored at −20°C, protected from light, until analysis. Alkaloid extracts were dissolved in dichloromethane and filtered with a 0.45 μm regenerated cellulose syringe filter before GC–MS analysis. The chromatographic analysis of the extracts was performed in a Thermo Scientific (Waltham, MA) Trace 1300, ISQ Single Quadrupole MS equipped with a TraceGOLD TG-5MS column (30 m × 0.25 mm × 0.25 μm) from Thermo Scientific. The oven temperature was kept at 150°C for 1 min, then increased at 5°C min^−1^ until 235°C and hold for 15 min, and further increased at 10°C min^−1^ until 280°C (held for 10 min). The injection volume was 1 μL and the split ratio of 1:5. The identification of the compounds was performed by the analysis of commercially available standards (gramine, (−)-sparteine, (−)-lupinine, lupanine; Sigma, St. Louis, MO) or by comparison with the NIST database (34). Quantification of individual alkaloids (mg kg^−1^ DM) was achieved from the calibration curves of standards prepared in dichloromethane analyzed under the same conditions as the samples. The total peak area was plotted as a function of concentration. Gramine, lupinine, sparteine, and lupanine were quantified as themselves. The other alkaloids were quantified as equivalents of the standard from the same chemical class (indole, piperidine, bicyclic, or tetracyclic quinolizidine).

### 2.5. Statistical analyses

Statistical analyses were performed with SAS software program (2022; Academic version, SAS Institute Inc., Carry, NC) using the General Linear Model and Linear Regression Model procedures. The statistical model used for residue biomass production and proportion included the fixed effects of species (*L. albus* ‘Estoril,’ *L. angustifolius* ‘Tango,’ *L. luteus* ‘Cardiga’), location (Mirandela, Vila Real), the species and location interaction, and the random residual error. For chemical composition, *in vitro* digestibility and alkaloids data, the model included the fixed effect of species (*L. albus* ‘Estoril,’ *L. angustifolius* ‘Tango,’ *L. luteus* ‘Cardiga’), biomass (straws, pod shells), location (Mirandela, Vila Real), and all double (species x biomass, species x location, biomass x location), and triple (species x biomass x location) interactions, and the random residual error. As the interaction species x biomass x location was never significant, it was removed from the model. Significance was set for *p*-values lower than 0.05 and multiple comparisons of means were carried out using the post-hoc Tukey test.

## 3. Results

### 3.1. Post-harvest biomass production

The production of straws was higher in *L. albus* ‘Estoril’ (3.10 t DM ha^−1^) and *L. luteus* ‘Cardiga’ (2.54 t DM ha^−1^) than *L. angustifolius* ‘Tango’ (1.34 t DM ha^−1^), no differences being observed between locations (*p* = 0.744; Table 1). Pod shells production followed a similar trend, being higher in white (1.00 t DM ha^−1^) and yellow (1.31 t DM ha^−1^) lupins and lower in narrow-leafed lupin (0.442 t DM ha^−1^). However, the production of pod shells harvested in Vila Real was nearly two-fold that obtained in Mirandela (1.17 and 0.666 t DM ha^−1^, respectively; Table 1).

**Table 1:** Post-harvest residue biomass production (t dry matter ha^−1^) and proportion (g kg^−1^) of total aerial biomass harvested (grains, straws, and pod shells) obtained from three European lupin species cultivated in two locations.

	Production			Proportion		
	Straws	Pod shells	Total residues	Straws	Pod shells	Total residues
Species						
*L. albus* ‘Estoril’	3.10^b^	1.00^b^	4.10^b^	511^b^	158^a^	669
*L. angustifolius* ‘Tango’	1.34^a^	0.442^a^	1.78^a^	543^b^	171^a^	715
*L. luteus* ‘Cardiga’	2.54^b^	1.31^b^	3.85^b^	437^a^	237^b^	674
Location						
Mirandela	2.27	0.666	2.94	546	160	706
Vila Real	2.38	1.17	3.56	449	217	666
Statistics						
*p*-values						
Species	0.001	<0.001	<0.001	0.004	<0.001	0.074
Location	0.744	<0.001	0.171	<0.001	<0.001	0.026
Species x Location	0.638	0.423	0.695	0.700	0.503	0.192
RSD	0.975	0.324	1.25	67.0	28.2	48.0
*R* ^2^	0.447	0.716	0.509	0.569	0.775	0.383
Adjusted *R*^2^	0.293	0.637	0.373	0.449	0.712	0.211

RSD, residual standard deviation; *R*^2^, coefficient of determination; ^a,b^ means within a column with different superscript letters are significantly different (*p* < 0.05).

Proportion of straws in the aerial biomass harvested (grains, straws, pod shells) were the highest in *L. albus* ‘Estoril’ (511 g kg^−1^) and *L. angustifolius* ‘Tango’ (543 g kg^−1^), while the proportion of pod shells was the lowest (158 and 171 g kg^−1^, respectively; Table 1). On the other hand, *L. luteus* ‘Cardiga’ produced less straws (437 g kg^−1^) and more pod shells (237 g kg^−1^) than the other two species (Table 1). The proportion of straws and pod shells in the aerial biomass differed between locations, the highest proportion being observed for straws in Mirandela, and for pod shells in Vila Real (Table 1).

### 3.2. Chemical composition

**Figure 1:**
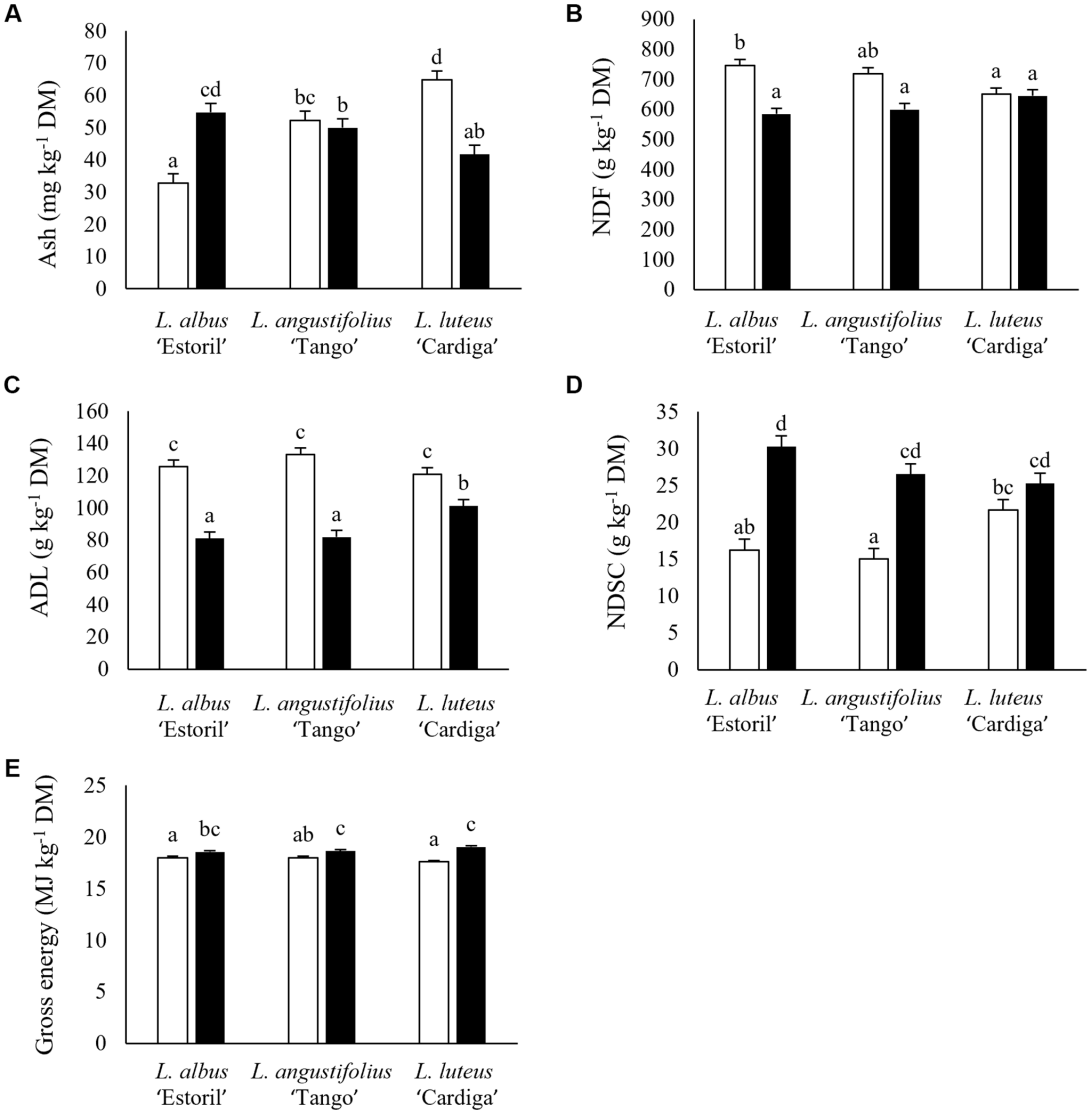
Ash **(A)**, neutral detergent fiber (NDF) **(B)**, acid detergent lignin (ADL) **(C)**, and neutral detergent soluble carbohydrates (NDSC) **(D)** content (mg kg^−1^ dry matter, DM), and gross energy (MJ kg^−1^ DM) **(E)** of post-harvest residue biomass obtained from three European lupin species. Straws, white bars; Pod shells, black bars. ^a,b,c,d^ means within each panel with different superscript letters are significantly different (*p* < 0.05).

**Figure 2:**
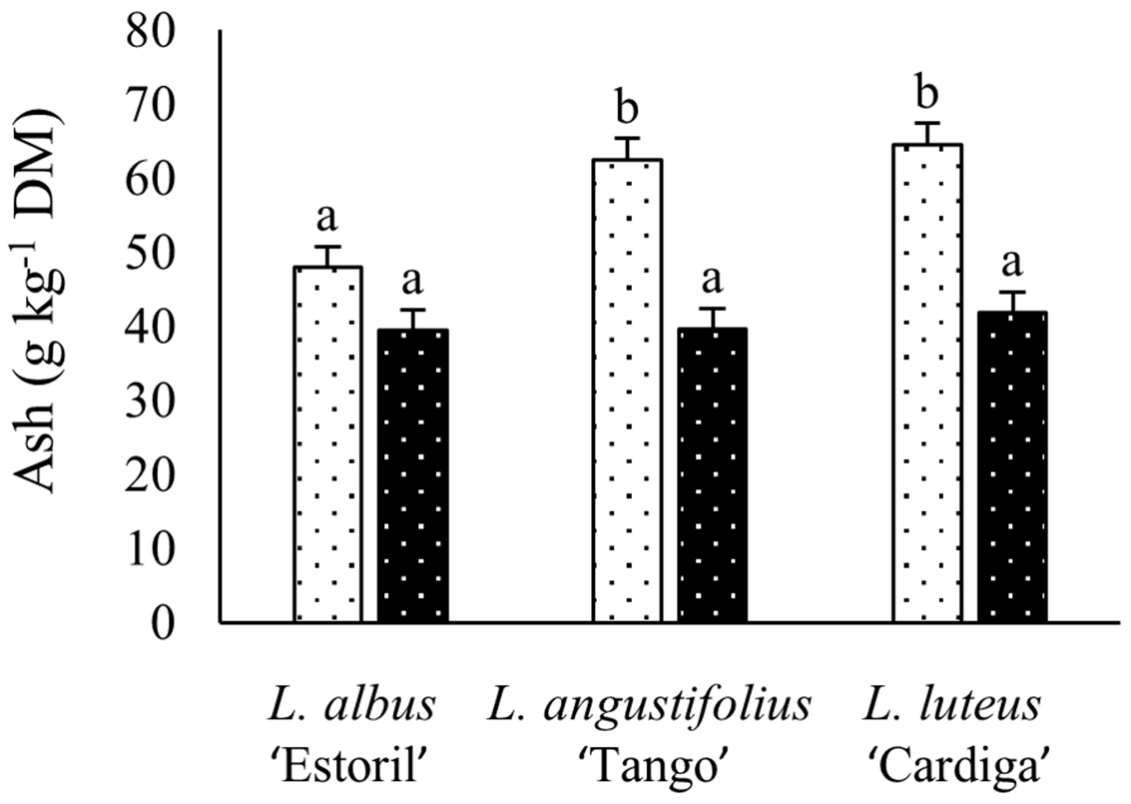
Ash content (g kg^−1^ dry matter, DM) of post-harvest residue biomass obtained from three European lupin species cultivated in two locations. Mirandela, white bars with black dots; Vila Real, black bars with white dots. ^a,b^ means with different superscript letters are significantly different (*p* < 0.05).

**Figure 3:**
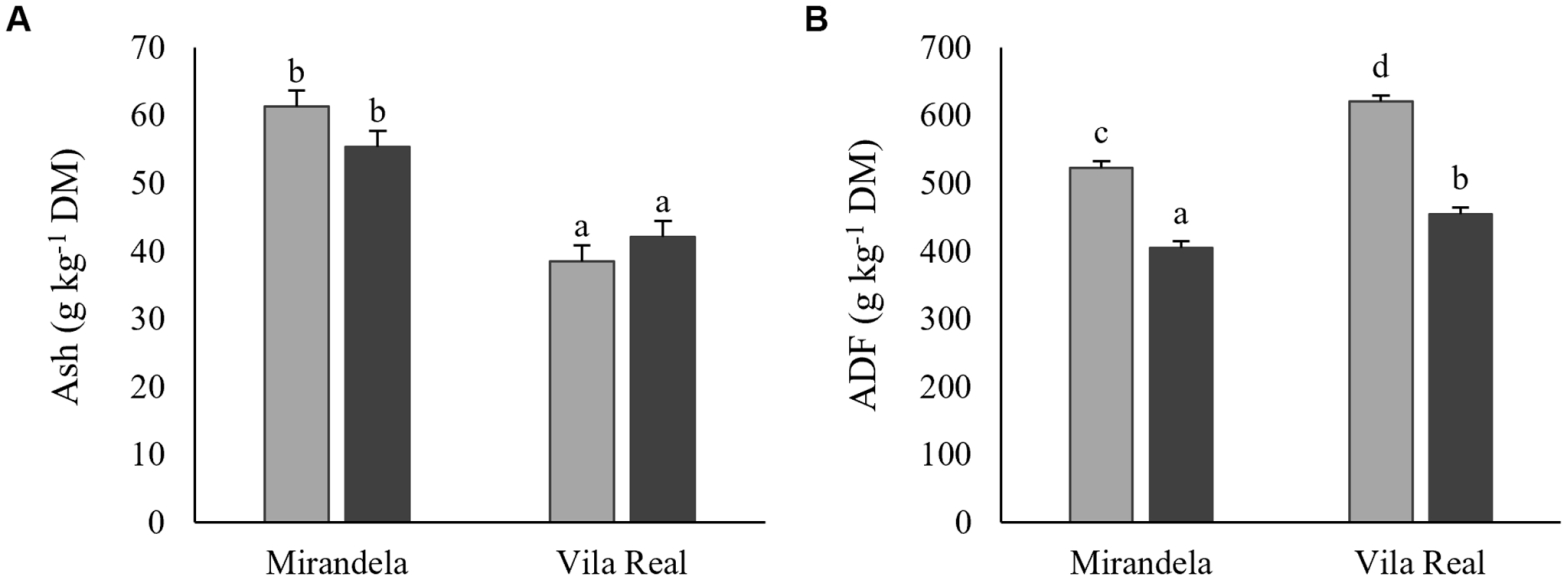
Ash **(A)** and acid detergent fiber (ADF) **(B)** content (g kg^−1^ dry matter, DM) of post-harvest residue biomass obtained from European lupin species cultivated in two locations (Mirandela and Vila Real). Straws, light gray bars; Pod shells, dark gray bars. ^a,b,c,d^ means within each panel with different superscript letters are significantly different (*p* < 0.05).

The chemical composition of post-harvest biomass residues is presented in Table 2. Ash content was the highest in *L. luteus* ‘Cardiga’ straws and the lowest in *L. albus* ‘Estoril’ straws (Figure 1A). Straws of *L. angustifolius* ‘Tango’ and *L. luteus* ‘Cardiga’ cultivated in Mirandela had higher ash than straws of *L. albus* ‘Estoril’ and all pod shells (Figure 2). In addition, straws and pod shells harvested in Mirandela presented higher ash content than those harvested in Vila Real (Figure 3A). The biomass residues of *L. angustifolius* ‘Tango’ presented higher CP content than that of *L. albus* ‘Estoril’ and *L. luteus* ‘Cardiga.’ Moreover, CP content of biomass residues harvested in Mirandela was higher than that harvested in Vila Real and tended to be affected by the interaction between species and location (*p* = 0.053) (Table 2). The EE content was only affected by biomass residue, with the pod shells having lower levels than straws (Table 2). The cell-wall constituents (NDF, ADF, and ADL) followed similar trends, being affected by biomass type, location, and the interaction between species and biomass type, for NDF and ADL, and the interaction between biomass and location for ADF (Table 2). The NDF content was the highest in straws of *L. albus* ‘Estoril’ and the lowest in pod shells of *L. albus* ‘Estoril’ and *L. angustifolius* ‘Tango,’ and in straws and pod shells of *L. luteus* ‘Cardiga’; *L. angustifolius* ‘Tango’ straws not differing from the others (Figure 1B). Lupin straws harvested in Vila Real presented the highest ADF content, followed by straws in Mirandela, pod shells in Vila Real and lastly by pod shells in Mirandela (Figure 3B). The ADL content was the highest in straws of all species, followed by pod shells of *L. luteus* ‘Cardiga,’ and the lowest in pod shells of *L. albus* ‘Estoril’ and *L. angustifolius* ‘Tango’ (Figure 1C). Non-structural carbohydrates (estimated as neutral detergent-soluble carbohydrates) content was the highest in *L. albus* ‘Estoril’ pod shells, which not differed from the other lupin species pod shells, and the lowest in *L. angustifolius* ‘Tango’ straws (Figure 1D). Gross energy content was the lowest in *L. albus* ‘Estoril’ and *L. luteus* ‘Cardiga’ straws and the highest in *L. angustifolius* ‘Tango’ and *L. luteus* ‘Cardiga’ pod shells (Figure 1E).

**Table 2:** Chemical composition (g kg^−1^ dry matter) and gross energy content (MJ kg^−1^ dry matter) of post-harvest residue biomass obtained from three European lupin species cultivated in two locations.

	Ash	CP	EE	NDF	ADF	ADL	NDSC	GE
Species								
*L. albus* ‘Estoril’	43.7^a^	53.0^a^	5.44	665	513	103	233	18.3
*L. angustifolius* ‘Tango’	51.1^b^	68.9^b^	5.51	659	500	107	208	18.3
*L. luteus* ‘Cardiga’	53.2^b^	58.3^a^	5.71	648	490	111	235	18.3
Biomass								
Straws	49.9	61.2	7.18	705	572	127	176	17.9
Pod shells	48.8	58.9	3.92	610	430	88.1	274	18.7
Location								
Mirandela	58.4	78.7	5.65	617	464	93.4	235	18.2
Vila Real	40.3	41.5	5.45	698	538	121	215	18.5
Statistics								
*p*-values								
Species	0.005	0.005	0.926	0.678	0.194	0.194	0.123	0.945
Biomass	0.621	0.531	<0.001	<0.001	<0.001	<0.001	<0.001	<0.001
Location	<0.001	<0.001	0.733	<0.001	<0.001	<0.001	0.090	0.010
Species x Biomass	<0.001	0.772	0.414	0.001	0.067	0.001	0.002	0.006
Species x Location	0.022	0.053	0.309	0.844	0.539	0.609	0.784	0.499
Biomass x Location	0.047	0.951	0.788	0.148	0.024	0.687	0.052	0.221
RSD	9.12	14.5	2.26	61.7	37.8	12.8	45.4	0.454
*R* ^2^	0.752	0.710	0.431	0.633	0.857	0.826	0.666	0.622
Adjusted *R*^2^	0.693	0.639	0.292	0.546	0.822	0.783	0.587	0.530

CP, crude protein; EE, ether extract; NDF, neutral detergent fiber; ADF, acid detergent fiber; ADL, acid detergent lignin; NDSC, neutral detergent soluble carbohydrates; GE, gross energy; RSD, residual standard deviation; *R*^2^, coefficient of determination; ^a,b^ means within a column with different superscript letters are significantly different (*p* < 0.05).

### 3.3. *In vitro* digestibility and metabolizable energy

**Figure 4:**
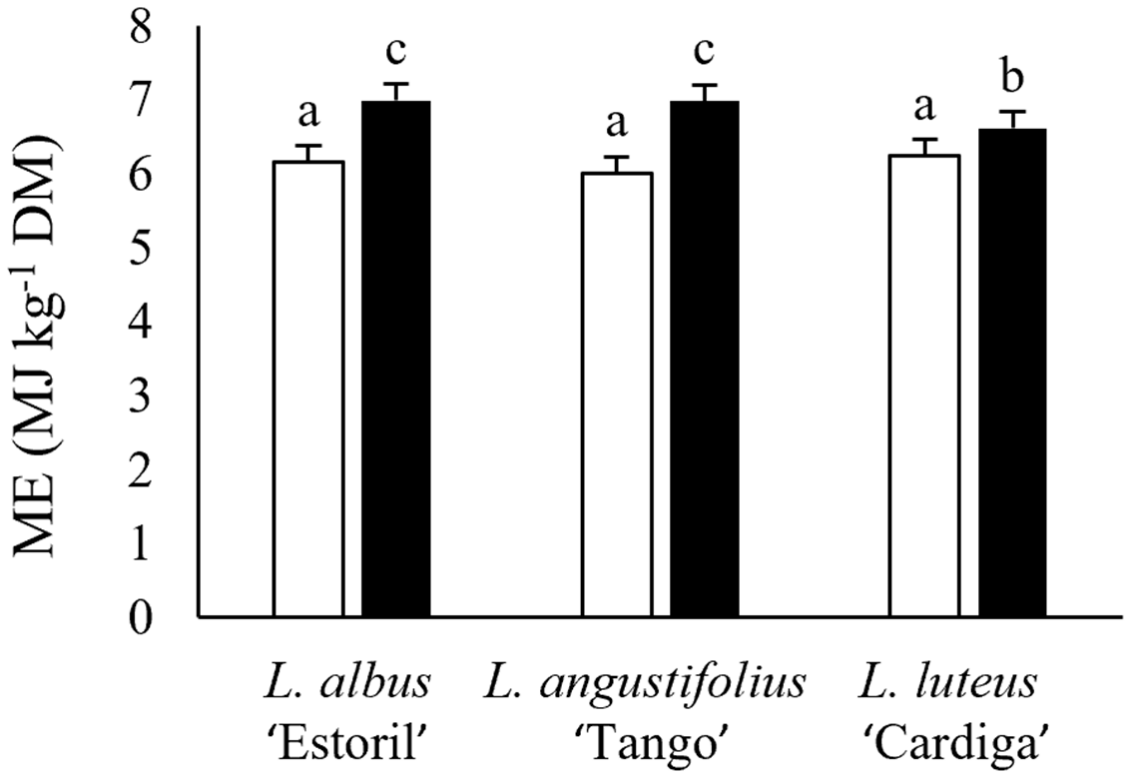
Estimated metabolizable energy (ME) content (MJ kg^−1^ dry matter, DM) of post-harvest residue biomass obtained from three European lupin species. Straws, white bars; Pod shells, black bars. ^a,b,c^ means within each panel with different superscript letters are significantly different (*p* < 0.05).

The *in vitro* digestibility was affected by the biomass type and the location (Table 3), with DMD and OMD of pod shells being higher than straws and the biomass residues produced in Mirandela being more digestible than those produced in Vila Real. The estimated ME content was affected by the interaction between species and biomass residue (Table 3 and Figure 4), being the highest in *L. albus* ‘Estoril’ and *L. angustifolius* ‘Tango’ pod shells, followed by *L. luteus* ‘Cardiga’ pods, and the lowest in straws regardless of the lupin species.

**Table 3:** Dry matter digestibility (DMD, %), organic matter digestibility (OMD, %) and estimated metabolizable energy (ME, MJ kg^−1^ dry matter) of post-harvest residue biomass obtained from three European lupin species cultivated in two locations.

	DMD	OMD	ME
Species			
*L. albus* ‘Estoril’	47.7	44.5	6.43
*L. angustifolius* ‘Tango’	49.2	46.6	6.58
*L. luteus* ‘Cardiga’	50.6	47.6	6.50
Biomass			
Straws	44.6	41.5	6.14
Pod shells	53.7	50.9	6.86
Location			
Mirandela	53.3	50.1	6.76
Vila Real	45.0	42.4	6.24
Statistics			
*p*-values			
Species	0.341	0.335	0.194
Biomass	<0.001	<0.001	<0.001
Location	<0.001	<0.001	<0.001
Species x Biomass	0.087	0.258	0.001
Species x Location	0.520	0.398	0.609
Biomass x Location	0.648	0.829	0.687
RSD	6.14	6.35	0.241
*R* ^2^	0.598	0.570	0.826
Adjusted *R*^2^	0.498	0.462	0.783

RSD, residual standard deviation; *R*^2^, coefficient of determination.

### 3.4. Alkaloids content

The GC–MS analysis of *Lupinus* sp. biomass residues extracts enabled the identification of 8 alkaloids (Supplementary Figure S1), distributed in three classes according to their chemical structure: indoles, piperidines and quinolizidines. Alkaloids identification was based on the comparison of the mass spectra (Supplementary Figure S2) with standards and with the NIST database (34). The main classes of alkaloids and total alkaloids content of lupin straws and pod shells are presented in Table 4. The individual indole, piperidine, bicyclic and tetracyclic quinolizidine alkaloids content is detailed in Table 5. No alkaloids were detected on *L. angustifolius* ‘Tango’ straws and pod shells.

**Table 4:** Main classes and total alkaloids (mg kg^−1^ dry matter) of post-harvest residue biomass obtained from three European lupin species cultivated in two locations.

	Indole	Piperidine	Quinolizidine			Total
			Bicyclic	Tetracyclic	Sum	
Species						
*L. albus* ‘Estoril’	nd	13.9	nd	8.32	8.32	22.2
*L. angustifolius* ‘Tango’	nd	nd	nd	nd	nd	nd
*L. luteus* ‘Cardiga’	41.0	17.4	116	34.1	150	209
Biomass						
Straws	21.1	17.6	66.0	10.4	43.4	71.5
Pod shells	61.0	13.7	166	32.1	115	159
Location						
Mirandela	60.7	20.4	153	26.2	103	154
Vila Real	21.3	10.9	79.0	16.2	55.7	77.2
Statistics						
*p*-values						
Species	–	0.148	–	<0.001	<0.001	<0.001
Biomass	<0.001	0.112	0.001	<0.001	<0.001	<0.001
Location	<0.001	<0.001	0.009	<0.001	0.002	<0.001
Species x Biomass	–	0.052	–	<0.001	<0.001	<0.001
Species x Location	–	0.810	–	0.961	0.012	0.006
Biomass x Location	0.048	0.005	0.792	0.348	0.717	0.684
RSD	21.7	7.72	58.5	5.98	45.0	61.4
*R* ^2^	0.715	0.508	0.604	0.931	0.841	0.836
Adjusted *R*^2^	0.644	0.390	0.505	0.914	0.802	0.796

RSD, residual standard deviation; *R*^2^, coefficient of determination; nd, not detected.

**Table 5:** Individual indole, piperidine, and bicyclic and tetracyclic quinolizidine alkaloids content (mg kg^−1^ dry matter) of post-harvest biomass obtained from three European lupin species cultivated in two locations.

	Indole	Piperidine			Bicyclic		Tetracyclic	
	Gramine	Smipine	Ammodendrine	Hydroxyammodendrine	Lupinine	Lusitanine	Sparteine	Lupanine
Species								
*L. albus* ‘Estoril’	nd	12.8	3.65	nd	nd	nd	4.60	3.97
*L. angustifolius* ‘Tango’	nd	nd	nd	nd	nd	nd	nd	nd
*L. luteus* ‘Cardiga’	41.0	nd	14.5	2.92	113	<12.7	34.1	nd
Biomass								
Straws	21.1	16.1	7.55	3.99	66.0	nd	7.78	5.66
Pod shells	61.0	9.51	8.37	<2.57	160	<12.7	30.9	<2.57
Location								
Mirandela	60.7	17.3	9.96	3.89	150	<12.7	22.9	6.96
Vila Real	21.3	8.31	5.96	<2.57	76.7	<12.7	15.8	<2.57
Statistics								
*p*-values								
Species	–	–	<0.001	–	–	–	<0.001	–
Biomass	<0.001	0.010	0.641	–	0.001	–	<0.001	–
Location	<0.001	0.001	0.028	–	0.008	–	<0.001	–
Species x Biomass	–	–	0.207	–	–	–	<0.001	–
Species x Location	–	–	0.100	–	–	–	0.086	–
Biomass x Location	0.048	0.262	0.077	–	0.834	–	0.046	–
RSD	21.7	5.31	5.62	2.19	56.9	2.85	5.61	2.80
*R* ^2^	0.715	0.609	0.697	0.437	0.595	0.275	0.945	0.721
Adjusted *R*^2^	0.644	0.512	0.624	0.296	0.493	0.154	0.932	0.652

RSD, residual standard deviation; *R*^2^, coefficient of determination; nd, not detected; <(value), detected below the limit of quantification.

**Figure 5:**
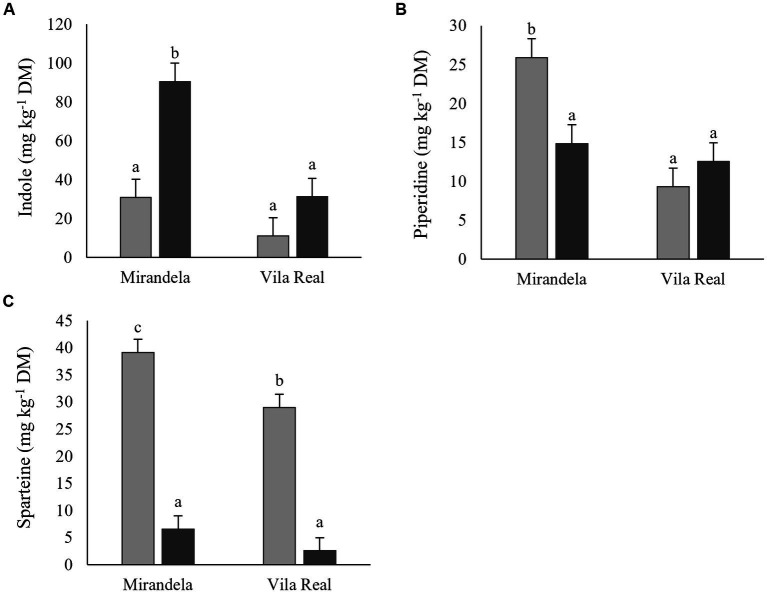
Indole (represented by gramine only) **(A)** and piperidine alkaloids **(B)**, and sparteine **(C)** content (mg kg^−1^ dry matter, DM) of post-harvest residue biomass obtained from European lupin species cultivated in two locations (Mirandela and Vila Real). Straws, light gray bars; Pod shell, dark gray bars. ^a,b^ means within each panel with different superscript letters are significantly different (*p* < 0.05).

**Figure 6:**
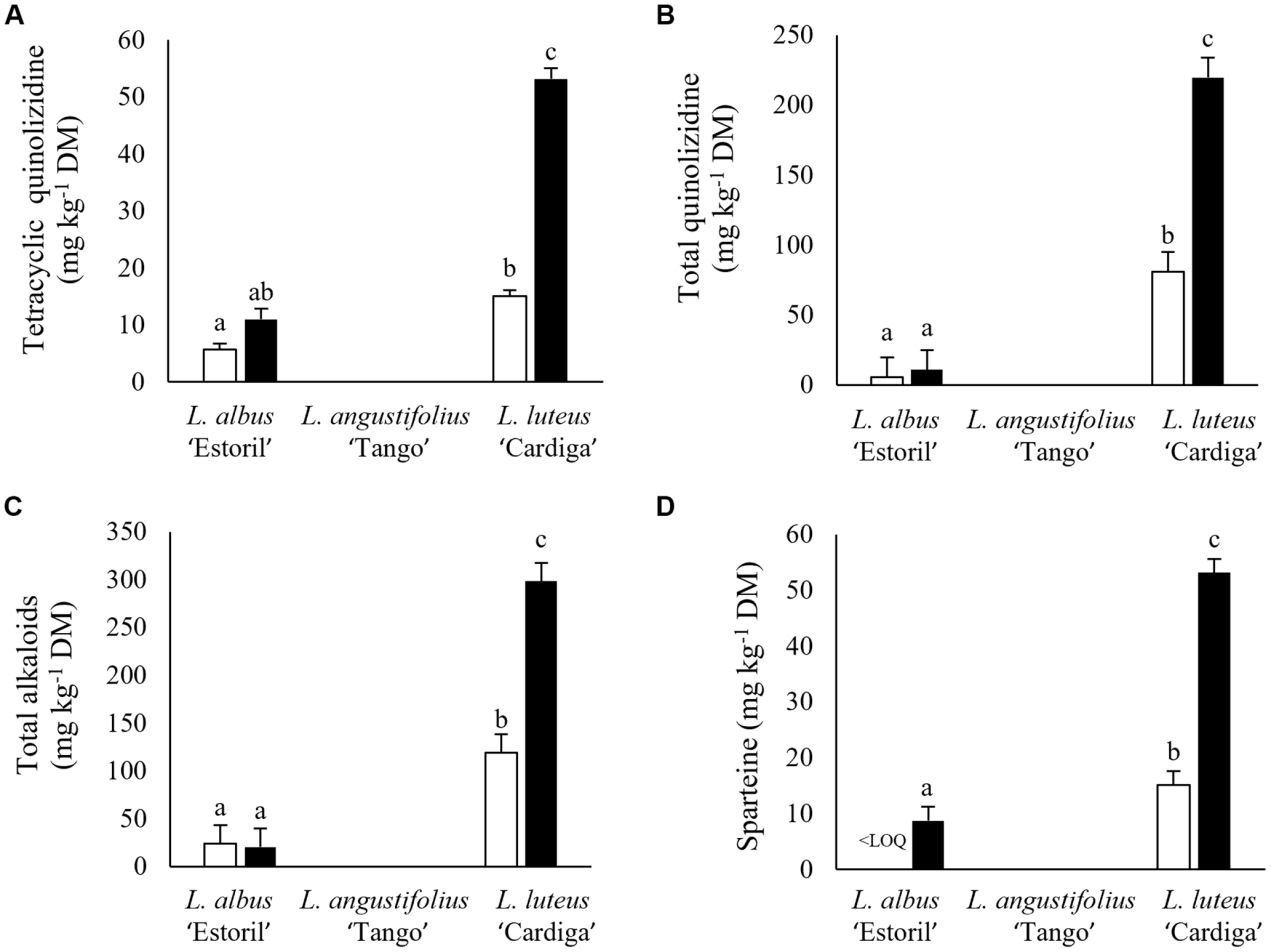
Tetracyclic quinolizidines **(A)**, total quinolizidines (bicyclic and tetracyclic) **(B)**, total alkaloids **(C)**, and sparteine **(D)** content (mg kg^−1^ dry matter, DM) of post-harvest residue biomass obtained from three European lupin species. Straws, white bars; Pod shells, black bars. ^a,b,c^ means within each panel with different superscript letters are significantly different (*p* < 0.05).

**Figure 7:**
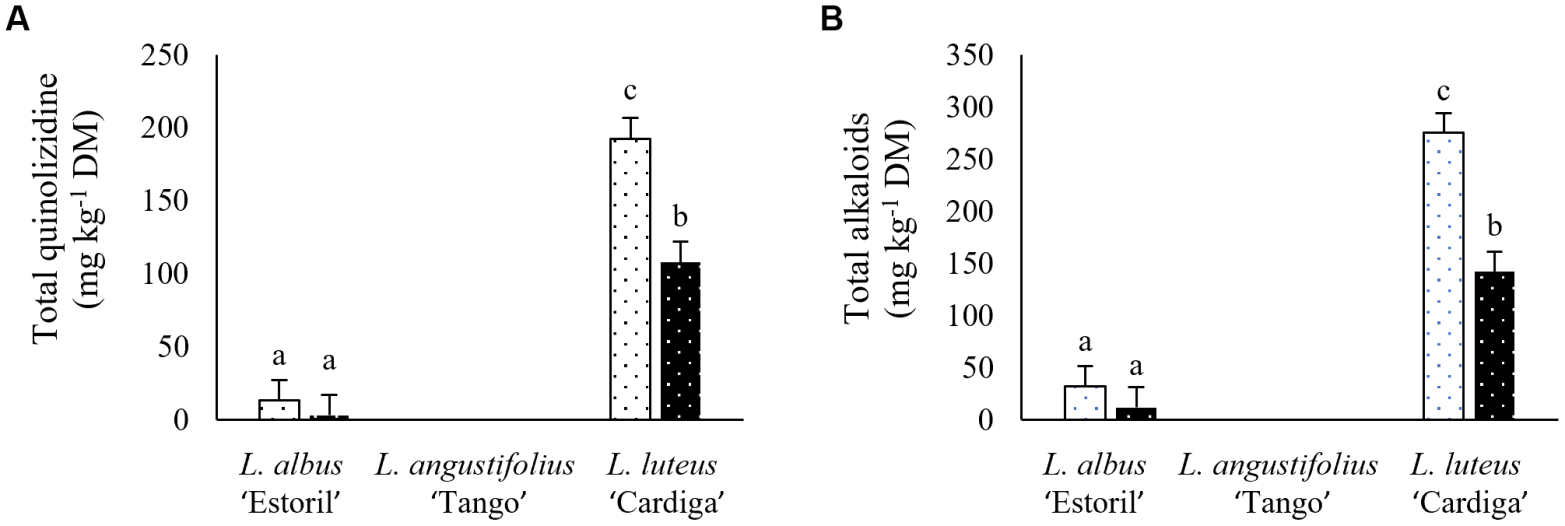
Total quiniolizidine alkaloids (bicyclic and tetracyclic) **(A)**, and total alkaloids **(B)** content (mg kg^−1^ dry matter, DM) of post-harvest residue biomass obtained from three European lupin species cultivated in two locations. Mirandela, white bars with black dots; Vila Real, black bars with white dots. ^a,b,c^ means with different superscript letters are significantly different (*p* < 0.05).

Gramine was the only indole alkaloid quantified, being exclusively present in *L. luteus* ‘Cardiga’ (Table 4). Indole content was similar in lupin straws harvested in Mirandela and straws and pod shells in Vila Real, while the content in pod shells harvested in Mirandela were nearly three folds higher than in Vila Real (Figure 5A). Piperidine alkaloids were higher in lupin straws harvested in Mirandela than in pods and straws harvested in Vila Real (Figure 5B). Bicyclic quinolizidine alkaloids were only detected in *L. luteus* ‘Cardiga,’ being higher in straws than pod shells and in residues harvested in Mirandela than in Vila Real (Table 4). Tetracyclic quinolizidine alkaloids content was the highest in *L. luteus* ‘Cardiga’ pod shells followed by straws, and the lowest in *L. albus* ‘Estoril’ straws; the content in *L. albus* ‘Estoril’ pod shells not differing from straws of ‘Estoril’ and ‘Cardiga’ (Figure 6A). In addition, tetracyclic quinolizidines were higher in lupin biomass residues harvested in Mirandela than in Vila Real (Table 4). The sum of quinolizidine alkaloids (bicyclic and tetracyclic) and total alkaloids content followed the same pattern, with *L. luteus* ‘Cardiga’ pod shells presenting the highest content, followed by ‘Cardiga’ straws, and the lowest content being found in *L. albus* ‘Estoril’ residues (Figures 6B,C). Similarly, biomass residues of *L. luteus* ‘Cardiga’ harvested in Mirandela presented the highest quinolizidine and total alkaloids content, followed by ‘Cardiga’ harvested in Vila Real, the lowest content being observed in *L. albus* ‘Estoril,’ regardless of location (Figures 7A,B). Smipine was only detected in *L. albus* ‘Estoril,’ being higher in straws than pod shells and in Mirandela than Vila Real (Table 5). Ammodendrine content was higher in pod shells than in straws and in biomass residues harvested in Mirandela than in Vila Real (Table 5). Hydroxyammodendrine was only detected in *L. luteus* ‘Cardiga,’ being quantified in straws harvested in Mirandela (Table 5). Lupinine was only detected in *L. luteus* ‘Cardiga,’ pod shells presenting higher content than straws, and biomass harvested in Mirandela with higher content than the obtained in Vila Real (Table 5). Lusitanine was detected in *L. luteus* ‘Cardiga’ pod shells, harvested in Mirandela and Vila Real, but below the limit of quantification. Sparteine content was the highest in straws harvested in Mirandela followed by those harvested in Vila Real, and the lowest in pod shells harvested in both locations (Figure 5C). In addition, pod shells of *L. albus* ‘Estoril’ presented the lowest sparteine content, followed by *L. luteus* ‘Cardiga’ straws, while the highest content was determined in *L. luteus* ‘Cardiga’ pod shells (Figure 6D); although detected in *L. albus* ‘Estoril’ straws, sparteine was below the limit of quantification. Lupanine was the only tetracyclic quinolizidine found in *L. albus* ‘Estoril’ straws harvested in Mirandela (Table 5).

The estimates for the maximum theoretical exposure of ruminant animals to main classes of alkaloids and total alkaloids of lupin post-harvest biomass residues is presented in Table 6. Considering an intake of residue biomass *ad libitum* and of 150 g protein-rich concentrate, a 55 kg body weight (BW) ram would be exposed to as much as 0.64 mg BW^−1^ total alkaloids if eating *L. albus* ‘Estoril’ harvested in Mirandela and 4.08 mg BW^−1^ total alkaloids if fed on *L. luteus* ‘Cardiga’ harvested in Mirandela. Similarly, with a DM intake of 2% of BW, of which 85% lupin biomass residues and 15% a protein-rich concentrate, a beef cow (500 kg BW) would be exposed to a maximum of 0.57 mg BW^−1^ total alkaloids if fed *L. albus* ‘Estoril’ harvested in Mirandela and 3.94 mg BW^−1^ total alkaloids when fed on *L. luteus* ‘Cardiga’ harvested in Mirandela.

**Table 6:** Estimates of maximum theoretical exposure to alkaloids for ruminant animals fed *Lupinus albus* ‘Estoril’ and *Lupinus luteus* ‘Cardiga’ post-harvest biomass-based diets (mixture of straws and pod shells in proportion of the dry matter produced per species by location).

Animal model	DMI (g kg^−1^ BW)	BDMI (g kg^−1^ BW)	Lupin species	Location	Alkaloid (mg kg^−1^ BW)					
					Indole	Piperidine	Quinolizidine			Total
							Bicyclic	Tetracyclic	Sum	
Merino rams (55 kg BW; straw *ad libitum* + 150 g concentrate)	21.6^1^	19.1	*L. albus* ‘Estoril’	Mirandela	–	0.41	–	0.23	0.23	0.64
	21.6^1^	19.1		Vila Real	–	0.18	–	0.05	0.05	0.23
	20.1^1^	17.6	*L. luteus* ‘Cardiga’	Mirandela	0.84	0.42	2.29	0.53	2.81	4.08
	20.1^1^	17.6		Vila Real	0.34	0.21	1.23	0.45	1.67	2.22
Beef cattle (500 kg BW; DMI = 2% BW; 15% of concentrate)	20.0	17.0	*L. albus* ‘Estoril’	Mirandela	–	0.37	–	0.21	0.21	0.57
	20.0	17.0		Vila Real	–	0.16	–	0.05	0.05	0.21
	20.0	17.0	*L. luteus* ‘Cardiga’	Mirandela	0.81	0.41	2.21	0.51	2.72	3.94
	20.0	17.0		Vila Real	0.33	0.20	1.18	0.43	1.62	2.15

BW, body weight; DMI, dry matter intake; BDMI, post-harvest biomass dry matter intake; ^1^according to Abreu and Bruno-Soares (21).

## 4. Discussion

The world growing population and the increasing demand for food are pressing the agricultural systems to evolve toward more efficient and sustainable practices to effectively address food and nutrient security (35). To achieve this societal challenge, the European stakeholders have adopted the strategy of Circular Economy and Bioeconomy (36, 37), focused on preventing and reducing waste and increasing its value through their incorporation into new processes (38). The agricultural sector produces a large amount of biomass throughout the agri-food chain, from farm to fork, discarded as waste, with negative economic, social, and environmental impacts (39). Global agri-food waste was estimated to contain nutrients to support food security for 2000 million people (40), highlighting the importance of agri-food biomass to a circular and low-carbon economy (41–43). As the ultimate up-cyclers, ruminants have the ability to convert agri-food residues, by-products, and co-products, or inedible to humans into foods of high biological value, thus addressing food security and environmental sustainability (44, 45). In this context, locally produced agricultural residues’ biomass can be used as ruminant feeds, thus effectively contributing to valorizing agricultural wastes and providing underexplored low-cost feed resources while promoting the circular economy.

The interest in native European legume production for food and feed has increased over the last few years. In 2021, European lupin production accounted for 391,342 tons, corresponding to 28.7% of the world production yield (13). Although scarce data exist on the agro-residues generated during lupin seed harvest and post-harvesting processes, it has been estimated that 7 tons of biomass residues are produced per ton of seed (46). Unlike cereal straws, lupin biomass residues (e.g., stalks, stubbles, straws, pod shells, husks) are traditionally left in the fields and burned in open fires and/or incorporated in the soil to promote carbon and minerals content (47). Alternatively, the residues can be grazed by livestock, particularly small ruminants, thus adding value to these biomasses with no environmental burden. However, grazing presents hazards as lupins may be colonized by several fungi causing important yield-limiting diseases, such as Phomopsis blights caused by *Diaporthe toxica* (48). When lupin stalks, stubbles, straws, and grains are left in the fields, in particular after the first rain, Phomopsis blights may become a health issue to livestock as *D. toxica* mycotoxins may induce lupinosis, a liver degenerative disease that can also cause brain damage and death (49).

Under this rationale, we anticipate that the biomass residues obtained from lupin grain harvest may be valorized if collected, stored, and used as fodder for ruminants in periods of feed scarcity. On the other hand, as legumes are harvested some centimeters off the ground, the stalks and stubbles that remain in the field could be further incorporated into the soil or processed as soil amendment (46), along with the root biomass that contributes to promote the soil OM and nutrient content (50). This holistic approach addresses the quest for biomass for soil amendment and animal feeding, pointing out the need to sustainably balance biomass demands (4).

Straws and pod shells comprise the main post-harvest biomass residues from lupin crop production. In this study, the production, nutritive value, and alkaloid content were assessed separately for straw and pod shells. Regardless of location, the combination of straws and pods per variety accounted for 63.5–74.3% of total aerial biomass harvested (grains, straws, and pod shells), agreeing with the biomass residues of *L. angustifolius* and *L. mutabilis* Sweet, also known as Andean lupin, which accounted, respectively, for 71.0 and 87.5% of total harvest (46, 47, 51). The overall results here presented suggest a poor adaptation of the early cultivar *L. angustifolius* ‘Tango’ to the specific climatic conditions of both studied locations during the agronomic year of 2018/2019. White, narrow-leafed, and yellow lupins are widely distributed in the Mediterranean region, being particularly adapted to acidic and sandy soils, with poor fertility and low water-holding capacity (52). However, the agricultural year was atypically hot, with the average maximum and minimum temperatures higher and with lower precipitation than the historical data, except for two months (November and April) that were cooler and rainier than the observed between 1971 and 2010, which may have posed particular issues to the drought-escaping *L. angustifolius* (53).

European lupin biomass residues have long been regarded as of low value. The lack of interest on legume biomass residues is reflected on the scarce availability of nutritive value data in the literature. Despite species-specific differences, the proximate composition of the three lupin species biomass residues evaluated confirmed their high fiber, moderate CP, and low EE content. However, when compared to cereal straws, white, narrow-leafed, and yellow lupins straws present higher CP, soluble carbohydrates, and lignin, and lower NDF content (19, 54). The ash and ADF content of biomass residues here evaluated were found to vary between sowing locations, reinforcing the impact of edaphoclimatic conditions on lupins production and composition of biomass residues. In contrast, no differences in NDF, ADF, and ADL content were observed in dry stems of *L. mutabilis* Sweet produced in winter Mediterranean and summer North European crop conditions (55).

To leverage the use of lupin biomass residues as feed resources, it is of utmost importance to evaluate their digestibility. *In vitro* DMD and OMD were similar among lupin species (47.7–50.6%) and higher in pod shells (53.7%) than in straws (44.6%). To the best of our knowledge, no data is available in the literature on lupin pod shells’ digestibility. Regarding straws, *L. albus* ‘Estoril’ presented lower DMD than the one reported by López (56) for *L. albus* sp. (42.1 vs. 69.3%), while that of *L. angustifolius* ‘Tango’ was higher than the one determined by Mulholland et al. (51) in sheep fed *L. angustifolius* sp. (47.5 vs. 53.7%). Differences in the nutritive value of lupin straws may be due to different varieties or cultivars, stage of harvest, stem-to-leaf ratio, and edaphoclimatic conditions (56). The latter is highlighted by the greater DMD and OMD of biomass residues harvested in Mirandela location than in Vila Real. In legume residues, the stem-to-leaf ratio is of particular importance as losses due to defoliation are quite high resulting in lupin straw mostly made up of stems, thus reducing their nutritive value (57). Therefore, leaf loss should be reduced to the minimum if lupin biomass residues are to be used as feed, harvesting as soon as maturity is achieved and using machinery that efficiently preserves the leaf component. Nutrient digestibility and bioavailability may also be limited by the phytochemicals present in lupins, including polyphenols, phytosterols, tocopherols, triterpenes, and alkaloids (58).

Traditional Mediterranean livestock systems use ancestral practices based on sustainability and circular economy that promote the use of locally available resources and the balance between agricultural practices, livestock production, environment, and household economy in an integrated approach. By revisiting these practices, the biomass residues of lupin grain production may be a valuable feed resource for ruminants. Considering the combined production of straws and pod shells per lupin species, the ME and CP content (per ha) of biomass residues accounted for, respectively, 26,123 MJ and 216 kg in *L. albus* ‘Estoril,’ 11,117 MJ and 124 kg in *L. angustifolius* ‘Tango’ and 24,560 MJ and 227 kg in *L. luteus* ‘Cardiga.’ Assuming an average ME requirement of dry and mid-pregnancy sheep with 50 kg BW of 9 MJ day^−1^ and that 85% of the requirements are provided by lupin biomass residues, the ME produced per ha here reported would be sufficient to maintain from 4 to 9 sheep for one year. Moreover, considering a diet with 10% CP (DM basis), the biomass residues could fulfill nearly half of sheep CP requirements.

To effectively assess the potential use of lupin biomass residues as animal feed, their alkaloid content was determined. Plants belonging to the genus *Lupinus* produce several alkaloids, including indoles, piperidines, and quinolizidines, which play important roles in defense mechanisms against predators and pathogens (59). Quinolizidines are the most abundant alkaloids in lupins, with over 170 bicyclic, tricyclic, and tetracyclic quinolizidines reported, being often referred to as lupin alkaloids (60, 61). Quinolizidine alkaloids are synthesized from lysine in the chloroplast, regulated by light and circadian rhythm, and then transported to leaves and other organs by the phloem, being accumulated in pods and then in seeds as they mature (22, 59). Alkaloid contents vary with species, cultivar and variety, environmental conditions, such as temperature, drought, soil conditions, location, and between years (59, 60, 62). Although these N secondary metabolites play important roles on plant defense mechanisms, they can also have toxic and teratogenic effects on ruminants. The teratogenic quinolizidine alkaloid anagyrine and the piperidine alkaloids ammodendrine and N-methylammodendrine have been described to cause crooked calf syndrome (63, 64), a disease induced by the consumption of the teratogenic lupin alkaloids during early pregnancy that causes maternal muscular weakness and ataxia as well as skeletal deformities in the newborn such as arthrogryposis, scoliosis, kyphosis, and cleft palate (65).

To the best of our knowledge, no study has yet assessed the alkaloids profile in white, narrow-leafed, and yellow lupin straws and pods. Although alkaloids content in stems, leaves, and pods decreases as they accumulate in seeds, *L. albus* ‘Estoril’ and *L. luteus* ‘Cardiga’ presented considerable amounts of alkaloids in straws (23.9 and 119 mg kg^−1^ DM) and pod shells (20.5 and 298 mg kg^−1^ DM). On the other hand, no alkaloids were detected in *L. angustifolius* ‘Tango’ biomasses. *Lupinus albus* ‘Estoril’ was characterized by the piperidine alkaloids smipine, followed by ammodendrine, and the tetracyclic quinolizidine lupanine. The bicyclic quinolizidine lupinine was the main alkaloid present in *L. luteus* ‘Cardiga,’ followed by the indole gramine, the tetracyclic quinolizidine sparteine, and the piperidine alkaloids ammodendrine and hydroxyammodendrine. Quinolizidine alkaloids of yellow lupin were at higher concentration in pod shells, which is in agreement with the late vigor of *L. luteus* (52), as a later seed maturation would be reflected in a longer transient accumulation of alkaloids in pods. Being an early vigor lupin species, no differences in alkaloid content were found between biomass residues in *L. albus* ‘Estoril.’ The impact of edaphoclimatic conditions on alkaloid content of straws and pod shells is also here unveiled. Indeed, lupin straw harvested in Mirandela presented higher piperidine alkaloids and the tetracyclic quinolizidine sparteine than straws harvest in Vila Real; no differences were found for pod shells alkaloids content. Conversely, indole alkaloids, represented by gramine alone, were detected at higher concentrations in pod shells harvested in Mirandela than in Vila Real, whereas straws presented lower and similar contents.

Few studies have evaluated the effects of lupin alkaloids on ruminants’ performance and feed intake and even fewer have assessed the maximum exposure level to these phytochemicals. Assuming Merino rams fed on lupin biomass residues *ad libitum* supplemented with a protein-rich concentrate (150 g day^−1^), with a daily DM intake of 21.6 g kg^−1^ BW, the maximum exposure to lupin alkaloids per day would be of 4.08 mg kg^−1^ BW with *L. luteus* ‘Cardiga’ biomass harvested in Mirandela. The exposure would be reduced to nearly half (2.22 kg^−1^ BW) if the same biomass was harvested in Vila Real. Much lower exposures to alkaloids would occur if sheep were fed *L. albus* ‘Estoril’ residues, achieving 0.64 mg kg^−1^ BW per day for biomasses harvested in Mirandela and 0.23 mg kg^−1^ BW per day for those harvested in Vila Real. No data was found in the literature on exposure limits in sheep or with alkaloids data that allowed the exposure estimation. However, *in vitro*, Aguiar and Wink (66) found that 1 mM sparteine or lupanine supplementation reduced gas production of hay after 24 h incubation with rumen inoculum collected from sheep fed a roughage-based diet, which corresponds to an exposure of 344 and 365 mg kg^−1^ BW per day for sparteine and lupanine, respectively, assuming an average DM intake of biomass residues for *L. albus* ‘Estoril’ and *L. luteus* ‘Cardiga’ (i.e., 20.85 g kg^−1^ DM per day) by 55 kg rams.

Estimations of the maximum exposure to lupin alkaloids were also calculated for beef cattle. For these animals, it was assumed a daily DM intake of 2% BW of a diet with 15% protein-rich concentrate. Maximum alkaloid exposure for beef cattle, per day, would be 3.94 mg kg^−1^ BW for *L. luteus* ‘Cardiga’ biomasses harvested in Mirandela and 2.15 mg kg^−1^ BW for those harvested in Vila Real. Daily exposures of 0.57 mg kg^−1^ BW and 0.21 mg kg^−1^ BW would be achieved if cattle were fed *L. albus* ‘Estoril’ biomass residues harvested in Mirandela and Vila Real, respectively. Data from three studies were used, one in heifers and two in dairy cows, to estimate the alkaloid exposure. Johnson et al. (67) found no effect on feed intake, weight gain, and feed efficiency of heifers fed a diet with *L. albus* ‘Tifwhite’-78 lupin seeds (193 g kg^−1^, DM basis) for 70 days, thus suggesting that alkaloids intake at 4.5 mg kg^−1^ BW per day were well tolerated by heifers (68). The effects of intact and heat-treated crushed *L. albus* seed supplemented at 150 and 300 g kg^−1^ (DM basis) on feed intake and milk yield were assessed in dairy cows (69); lupanine and 13-hydroxylupanine being the only alkaloids determined. Voluntary feed intake and milk yield were reduced with high alkaloid supplementation and when cows were fed intact seeds compared to heat treated seeds, being the maximum exposure tolerated by dairy cows calculated by Schrenk et al. (68) as 5.2 mg lupanine and 13-hydroxylupanine kg^−1^ BW day^−1^. On the other hand, Engel et al. (70) reported no negative effect of feed intake, milk yield and milk fat and protein composition in dairy cows fed 1774 mg *L. angustifolius* quinolizidine alkaloids day^−1^, while at 3548 mg day^−1^ milk yield, but not feed intake and milk composition, was reduced. Assuming a BW of 650 kg, we may assume impaired milk yield at 5.45 mg quinolizidine alkaloids kg^−1^ BW day^−1^.

Considering all data available, no negative effects are expected on feed intake, rumen fermentation, and performance of sheep and cattle fed on lupin biomass residues, thus highlighting their potential as alternative forage. However, future studies should be conducted *in vivo* to validate the safety of lupin biomass residues as alternative feeds for ruminant animals as well as alkaloids maximum exposure. These studies are further supported by the detection of quinolizidine alkaloids in the milk of dairy cows fed as low as 2.73 mg lupin seed kg^−1^ BW per day (assuming 650 kg BW) (70), highlighting the need to evaluate the potential transfer of alkaloids from dietary lupin biomass residues to dairy cows and sheep milk as it may pose toxicological concerns to consumers (70).

## 5. Conclusion

The present study addresses a poorly investigated topic, the use of undervalued lupin post-harvest biomass residues on ruminant feeding as a strategy to leverage the sustainability of production systems and ecological synergies between crop byproducts and animal management under a circular economy approach. Although species-specific traits were found, the native European lupin species, *L. albus* ‘Estoril,’ *L. angustifolius* ‘Tango,’ and *L. luteus* ‘Cardiga,’ biomass residues (straws and pod shells) have considerable production yield and higher nutritive value than cereal straws. Despite their alkaloid content, particularly of *L. luteus* ‘Cardiga,’ it is anticipated that lupin post-harvest biomass residues present no constraint for sheep and cattle even when consumed *ad libitum*. Overall results highlight the potential of *Lupinus* sp. biomass residues as alternative feeds for ruminant animals. As lupin production is estimated to continue to grow in Europe and worldwide, increasing the availability of post-harvest biomass residues and upcycling as feed for ruminants leverages its value and contribution to a more sustainable European farming system, being of particular importance in time of fodder shortage or scarcity in times of climate change.

## Data availability statement

The original contributions presented in the study are included in the article/Supplementary material, further inquiries can be directed to the corresponding author.

## Ethics statement

The rumen contents collection procedure was reviewed and approved by the Animal Ethics Committee of the School of Medicine and Biomedical Sciences, University of Porto, licensed by the Portuguese General Directorate for Food and Veterinary (permit #0421/000/000/2021), and performed by trained scientists (FELASA category C).

## Author contributions

HT conceived and designed the field study. AM, CM, and CC conducted the field study and sample collection. AF and HT conceived and designed the experimental study. MM, IV, and CS conducted the analytical work. PC was involved in animal manipulation and sampling. MM, IV, and AF performed the statistical analysis. MM, IV, CS, AC, and AF analyzed the data. MM and AF wrote the first draft of the manuscript. All authors contributed to manuscript revision, read, and approved the submitted version.

## Funding

This work received financial support from AgriFood XXI – Development and consolidation of research in the agrifood sector in Northern Portugal I&D&I project (NORTE-01-0145-FEDER-000041), co-financed by European Regional Development Fund through NORTE 2020 programme (Programa Operacional Regional do Norte 2014/2020), and from Portuguese Foundation for Science and Technology (FCT/MCTES) through projects UIDB/04033/2020, UIDB/50006/2020, and UIDP/50006/2020. MM and IV acknowledge FCT for funding through program DL 57/2016 – Norma transitória (Ref. SFRH/BPD/70176/2010 and SFRH/BPD/111181/2015, respectively), and CS acknowledge AgriFood XXI project for the contract.

## Conflict of interest

The authors declare that the research was conducted in the absence of any commercial or financial relationships that could be construed as a potential conflict of interest.

## Publisher’s note

All claims expressed in this article are solely those of the authors and do not necessarily represent those of their affiliated organizations, or those of the publisher, the editors and the reviewers. Any product that may be evaluated in this article, or claim that may be made by its manufacturer, is not guaranteed or endorsed by the publisher.
