# Supplementary material for: Upcycling post-harvest biomass residues from native European Lupinus species: from straws and pod shells production to nutritive value and alkaloids content for ruminant animals
Source: Front Nutr. 2023 Jul 13;10:1195015. doi: 10.3389/fnut.2023.1195015 (PMC10374839; doi:10.3389/fnut.2023.1195015)
Supplement: Supplementary file 1 [file Data_Sheet_1.pdf]

frontiers

Supplementary Material

Upcycling post-harvest biomass residues from native European *Lupinus* species: from straws and pod shells production to nutritive value and alkaloids content for ruminant animals

Margarida R. G. Maia*, André Monteiro, Inês M. Valente, Carla Sousa, Carla Miranda, Carlos Castro, Paulo P. Cortez, Ana R. J. Cabrita, Henrique Trindade, António J. M. Fonseca

*** Correspondence:** mrmaia@icbas.up.pt

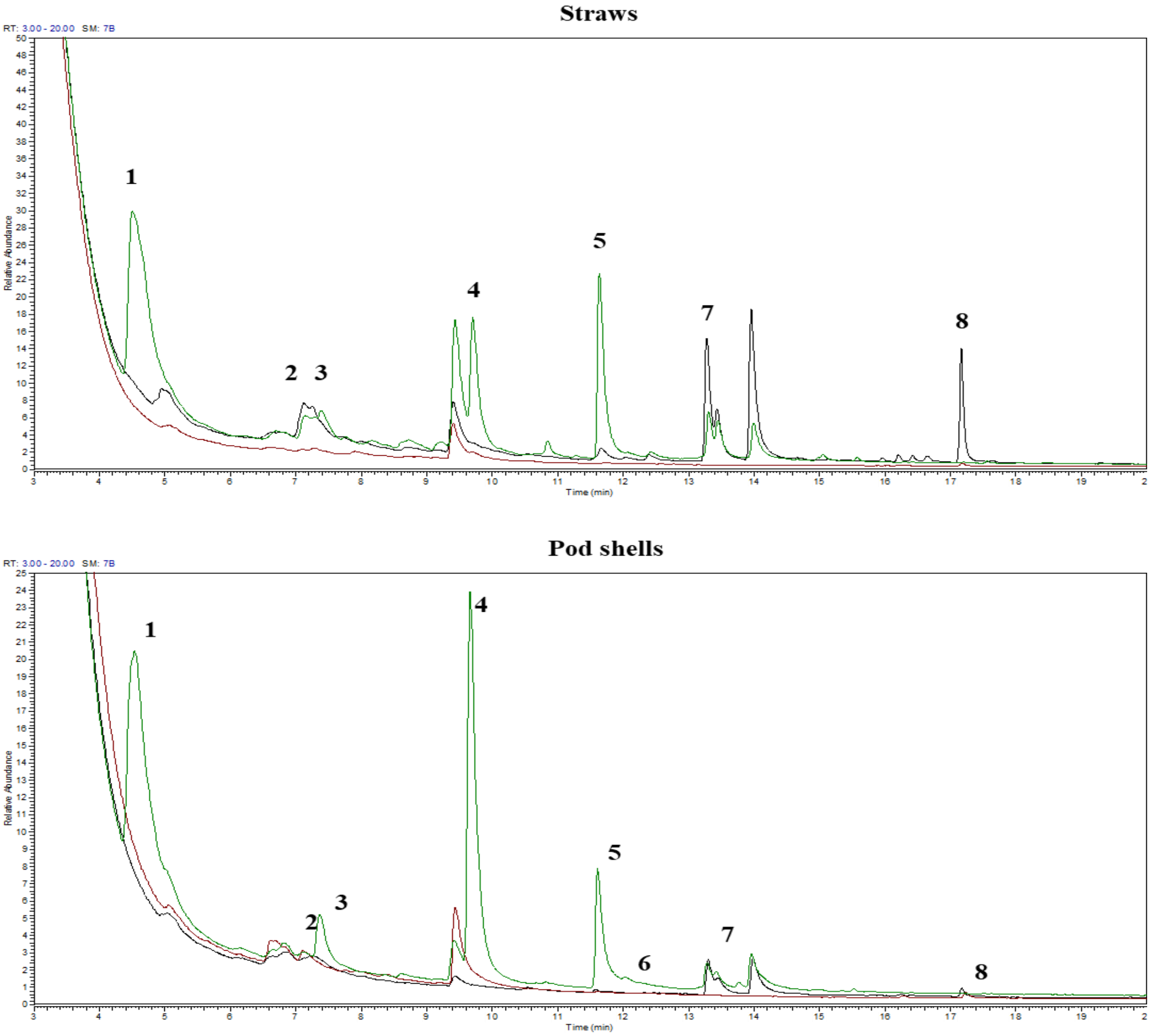

Supplementary Figure S1. Total ion GC-MS chromatograms of the straws and pod shells extracts of the three Lupinus species: *L. albus* 'Estoril' (black), *L. angustifolius* 'Tango' (red), and *L. luteus* 'Cardiga' (green). (1) Lupinine, (2) smipine, (3) gramine, (4) sparteine, (5) ammodendrine, (6) lusitanine, (7) hydroxyammodendrine, and (8) lupanine. Mass spectra for each alkaloid is presented in Supplementary Figure S2.

(1) Lupinine

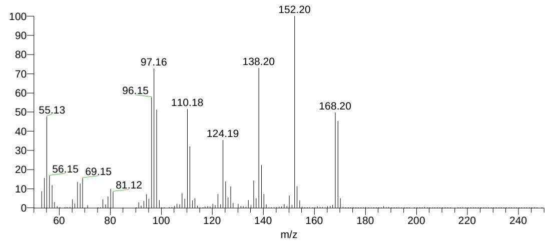

(2) Smipine

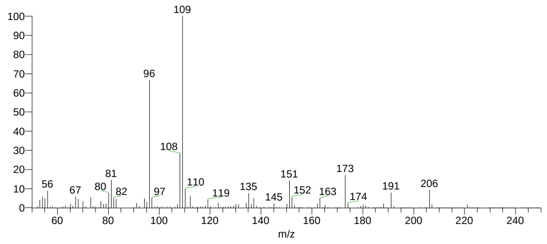

(3) Gramine

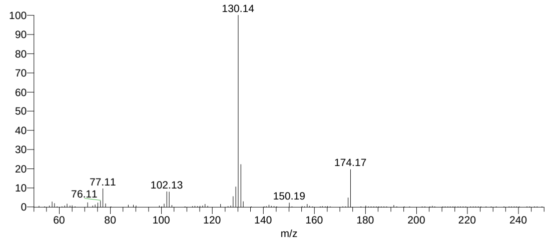

(4) Sparteine

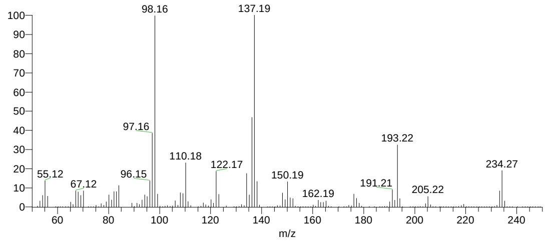

(5) Ammodendrine

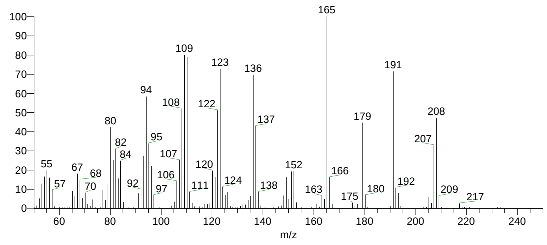

(6) Lusitanine

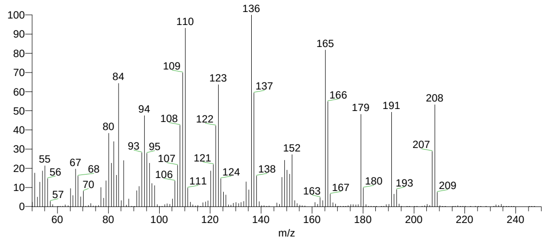

(7) Hydroxyammodendrine

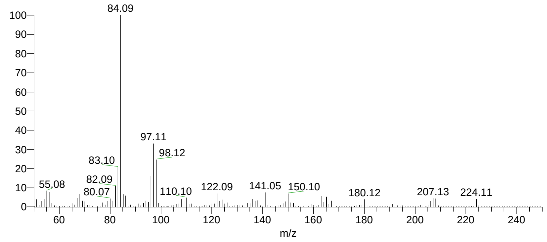

(8) Lupanine

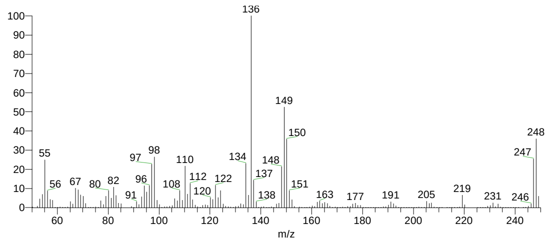

Supplementary Figure S2. Mass spectra (electron ionization) of the identified alkaloids in *Lupinus* sp. straws and pod shells.
